# Role of IL-10-Producing Natural Killer Cells in the Regulatory Mechanisms of Inflammation during Systemic Infection

**DOI:** 10.3390/biom12010004

**Published:** 2021-12-21

**Authors:** Israel Martinez-Espinosa, José A. Serrato, Blanca Ortiz-Quintero

**Affiliations:** Department of Research in Biochemistry, Research Unit, Instituto Nacional de Enfermedades Respiratorias Ismael Cosío Villegas, Mexico City 14080, Mexico; imartinezfq@gmail.com (I.M.-E.); serratoiner@gmail.com (J.A.S.)

**Keywords:** IL-10, natural killer cells, inflammation, systemic infections

## Abstract

Natural killer (NK) cells have the dual ability to produce pro-inflammatory (IFNγ) and anti-inflammatory (IL-10) cytokines during systemic infection, which points to their crucial role both as inflammatory effectors for infection clearance and as regulators to counterbalance inflammation to limit immune-mediated damage to the host. In particular, immunosuppressive IL-10 secretion by NK cells has been described to occur in systemic, but not local, infections as a recent immunoregulatory mechanism of inflammation that may be detrimental or beneficial, depending on the timing of release, type of disease, or the infection model. Understanding the factors that drive the production of IL-10 by NK cells and their impact during dualistic inflammatory states, such as sepsis and other non-controlled inflammatory diseases, is relevant for achieving effective therapeutic advancements. In this review, the evidence regarding the immunoregulatory role of IL-10-producing NK cells in systemic infection is summarized and discussed in detail, and the potential molecular mechanisms that drive IL-10 production by NK cells are considered.

## 1. Introduction

An adequate and precise balance between pro-inflammatory and anti-inflammatory immune responses is required to effectively eliminate infectious pathogens while limiting immune-mediated damage to the host. This complex balance is particularly relevant in systemic infection when infection with pathogens that have not been contained can lead to a dysregulated systemic inflammatory response, leading to severe host pathology and high morbidity and mortality.

Natural killer (NK) cells are innate cytotoxic lymphocytes recently classified as innate lymphoid cells that have a natural cytolytic ability against virus-infected cells and malignant cells [1]. NK cells play an essential role in the early control and pathogen clearance of viral and microbial infections as well as in tumor immunosurveillance in humans and mice. The conventional effector functions of NK cells include their cytotoxic activity and the production of pro-inflammatory cytokines, such as interferon gamma (IFNγ), which induce a potent inflammatory response [2]. However, NK cells are also able to dampen immune responses to several pathogens by producing the anti-inflammatory cytokine interleukin-10 (IL-10) during systemic infection [3]. The latter function can be beneficial to the host by protecting it from uncontrolled immune-mediated pathology of tissue and organs, or it can be detrimental to the host through the promotion of immunosuppression and, consequently, pathogen persistence and spread. This dual function of NK cells and the factors that influence their specific effect during immune responses should be revised to elucidate the complex regulatory mechanisms involved in normality and immune diseases. The immunoregulation of inflammatory response is particularly relevant in systemic infection diseases, such as sepsis, where an uncontrolled inflammatory response results in an immunopathology that is potentially fatal to the host.

Importantly, evidence suggests that IL-10 production by NK cells is induced during disseminated, but not during locally restricted, infections [4]. Perona-Wright et al. [4] found that NK cells were the main source of IL-10 during acute systemic infection with the protozoan *Toxoplasma gondii*, the Gram-negative bacterium *Yersinia pestis*, and Gram-positive *Listeria monocytogenes*, suggesting that the production of IL-10 by NK cells in response to systemic inflammation is independent of the pathogen. By contrast, localized infection with intranasal instillation of influenza virus and the attenuated *Yersinia pestis* strain resulted in NK cells producing IFNγ but not IL-10 [4].

**Figure 1:**
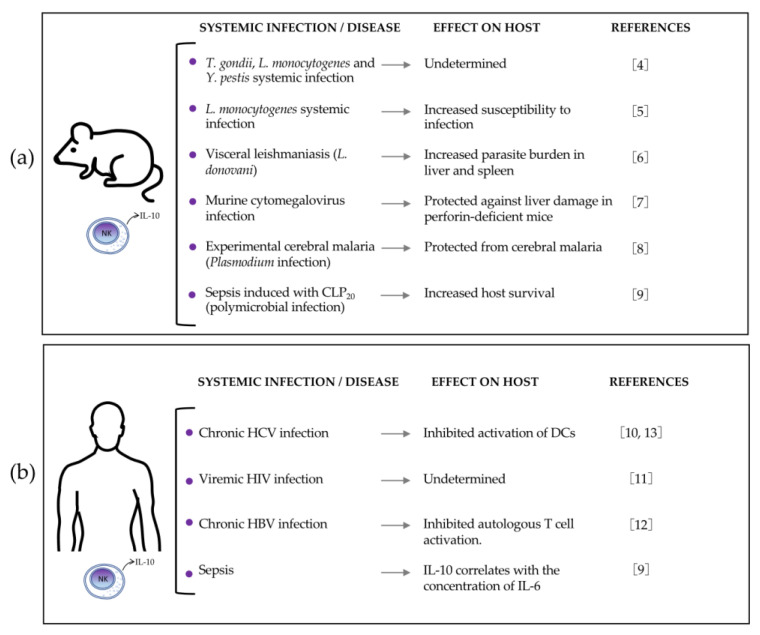
Natural killer (NK) cells produce interleukin-10 (IL-10) during diverse systemic infection models and some diseases in humans. The figure shows the (**a**) mouse models, and (**b**) human diseases in which NK cells are main producers of IL-10, and the observed effect on the host. CLP—cecal ligation and puncture; CLP20—cecal ligation and puncture with 1 puncture; HCV—hepatitis C virus; HBV—hepatitis B virus; HIV—human immunodeficiency virus; DCs—dendritic cells.

Recent evidence has indicated that NK cells are the main cell type producing IL-10 during diverse systemic infectious models in mice and several chronic diseases in humans (Figure 1). *Listeria monocytogenes* (Lm) systemic infection and experimental visceral leishmaniasis are the two animal models that have shown a detrimental effect of IL-10 produced by NK cells on immune host resistance against the pathogen [5,6]. On the other hand, murine cytomegalovirus (MCMV) infection, experimental cerebral malaria (ECM), and sepsis induced by cecal ligation and puncture (CLP) are the three animal models of systemic infection in which a beneficial immunoregulatory effect of IL-10 produced by NK cells on the host has been reported [7,8,9]. In human diseases, the presence of IL-10-producing NK cells has been reported in chronic hepatitis C virus (HCV) infection, viremic human immunodeficiency virus (HIV) infection, chronic hepatitis B virus (HBV) infection, and sepsis [9,10,11,12] (Figure 1). Some studies in humans have reported a detrimental role of IL-10-producing NK cells in suppressing host resistance against the pathogen [12,13].

The following sections describe the published evidence regarding the immunoregulatory role of IL-10-producing NK cells in systemic models of infection and human disease as a protective or deleterious factor in the host immune response. The potential molecular mechanisms that drive IL-10 production by NK cells are also discussed.

## 2. Role of IL-10-Producing NK Cells in Models of Systemic Infection

### 2.1. IL-10-Producing NK Cells during Listeria Monocytogenes (Lm) Systemic Infection

Lm is a Gram-positive intracellular bacterial pathogen that often contaminates food and causes infection, with different levels of severity. Most people who ingest the contaminated food develop mild gastrointestinal symptoms; however, in some individuals, Lm causes severe systemic infection and is propagated to peripheral organs (listeriosis), which often proves fatal. Although the incidence of severe listeriosis is low, Lm infections are a public health issue, owing to the frequency of outbreaks associated with contaminated food. The factors involved in the increased susceptibility of some individuals to severe infections are poorly understood, but systemic infections are mostly reported in the elderly, pregnant women, and immunocompromised individuals [14,15,16]. Most of the knowledge regarding pathogenesis and immune response to Lm has been obtained from the murine model of listeriosis. In this animal model, systemic infection with Lm leads to an innate immune response characterized by a cytotoxic T lymphocyte response, the formation of Th1 effector cells, and the production of the pro-inflammatory cytokine IFNγ by activated NK cells and memory T cells [17,18,19]. However, Perona-Wright et al. [4] showed in 2009 that most circulating NK cells produce the anti-inflammatory cytokine IL-10 during systemic Lm infection. Using *Il10*-eGFP-reporter mice (VertX, with genetic background C57BL/6J), the authors observed that 79% of blood NK cells became GFP-positive during acute infection with this bacterium.

**Figure 2:**
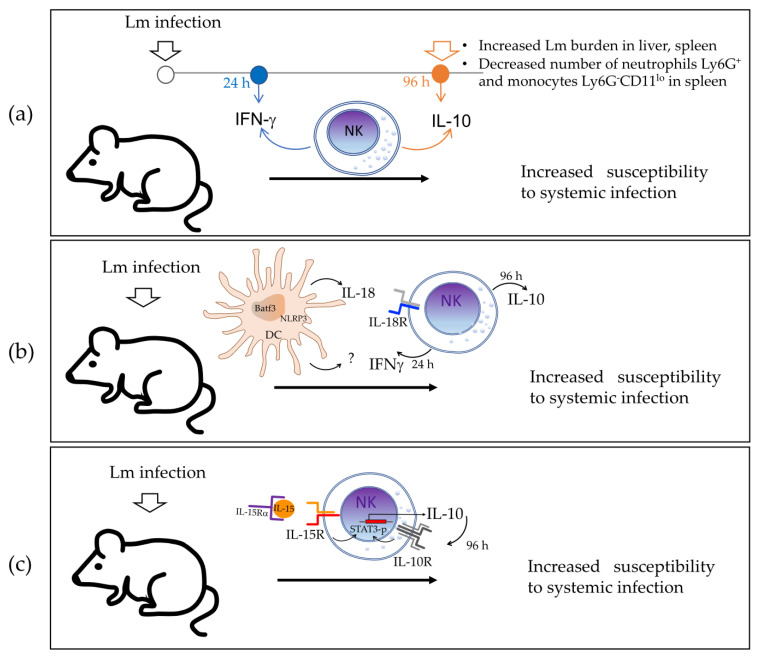
Proposed models of the role of IL-10-producing NK cells in *Listeria monocytogenes* (Lm) systemic infection in mice. (**a**) In this first study, it was found that NK cells produce pro-inflammatory IFNγ during the early phase of infection (24 h post-infection) but rapidly switch to anti-inflammatory IL-10 production and become the main source of IL-10 (96 h post-infection). IL-10-deficient mice that received NK cells from wild-type mice showed an increased Lm burden in the liver and spleen and a reduced number of neutrophils and monocytes in the spleen compared with those receiving IL-10-deficient NK cells, suggesting that IL-10 produced by NK cells increases susceptibility to systemic bacterium infection. (**b**) In this study, the kinetic of IFNγ and IL-10 production by NK cells during Lm systemic infection was confirmed. In addition, IL-18 produced by DCs expressing NLRP3 and Batf3 is required but is not sufficient to induce IL-10 production by NK cells in infected mice. (**c**) In this study, it was discovered that IL-15 signaling induces early STAT3 activation in NK cells required for the IL-10 production response to Lm infection. This, in turn, feeds back through IL-10R in NK cells to promote sustained STAT3 activation and IL-10 production by NK cells observed at 96 h post-infection, which is associated with increased susceptibility to Lm systemic infection. Lm—Listeria monocytogenes; IFNγ—interferon gamma; DCs—dendritic cells; NLRP3—NOD-, LRR-, and pyrin domain-containing protein 3; Batf3—basic leucine zipper transcriptional factor ATF-like 3; IL-10R—IL-10 receptor; IL-18R—IL-18 receptor; IL-15R—IL-15 receptor.

Later in 2016, Clark et al. [5] found that NK cells produce IFNγ during the early phase of infection but rapidly switch to IL-10 production. Their results also suggested that IL-10 produced by NK cells increases susceptibility to systemic bacterium infection by reducing the number of Ly6G^+^ neutrophils and Ly6G^−^CD11^lo^ monocytes in the spleen of infected mice (Figure 2a). They assessed IL-10 production using the tiger IL-10 GFP-reporter mouse model (where the 3′UTR of IL-10 remains unaltered) and found that GFP staining was selectively increased in NK cells from the spleen, liver, and blood of Lm-infected mice at 72 h post-infection, whereas the IL-10 GFP-reporter expression was not observed at 24 h post-infection. Intracellular staining for IFNγ showed that most NK cells indeed produce this pro-inflammatory cytokine at 24 h post-infection, but its levels declined by 96 h, which suggests the time-regulated dual function of NK cells during Lm systemic infection. The authors demonstrated that NK cells were the main source of IL-10 by performing adoptive transfer experiments in CD45.2^+^ or CD45.1^+^ B6.*il10*^−/−^ recipients that were infected with Lm and then received NK cells transferred from wild-type (wt) CD45.1 (B6) or from IL-10-deficient CD45.2 (B6.*il10*^−/−^) mice. Results indicated that protein lysates of splenocytes from the B6.*il10*^−/−^ recipients of NK cells from wt but not from IL-10-deficient mice contained IL-10 protein at 96 h post-infection. In addition, at 96 h post-infection, the B6.*il10*^−/−^ mice that received wt NK cells showed an increased Lm burden in the liver and spleen (10- to 100-fold), compared with those that received IL-10-deficient NK cells, suggesting that IL-10 produced by NK cells increases susceptibility to systemic bacterium infection. The authors suggested that IL-10-producing NK cells may suppress the accumulation and activation of inflammatory myeloid cells based on their finding of an increased number of neutrophils Ly6G^+^ and monocytes Ly6G^−^CD11^lo^ in the spleen of infected B6.*il10*^−/−^ recipients, which was reduced when mice were transferred with wt NK cells capable of producing IL-10.

Clark et al. [20] reported in 2018 that the cytokine IL-18 promotes IL-10 production by NK cells in the murine model of systemic Lm infection, which is dependent on the expression of NOD-, LRR-, and pyrin domain-containing protein 3 (*Nlrp)3* and the basic leucine zipper transcriptional factor ATF-like 3 (*Batf3*) by dendritic cells (DCs). The authors used in vivo models of Lm-infected B6.*Il18^−/−^*, B6.*Il18r^−/−^*, B6.*Nlrp3*^−/−^, and B6.*Batf3*^−/−^ mice in which they found almost undetectable serum IL-10 and reduced Lm burdens in the spleen and liver compared to B6 mice at 72 h post-infection, showing the relevance of IL-18, IL-18R1, NLRP3, and Batf3 for IL-10 production and resistance to Lm systemic infection. In addition, compared with B6 mice, Lm-infected B6.*Il18*^−/−^, B6.*Nlrp3*^−/−^, and B6.*Batf3*^−/−^ mice showed reduced levels of serum IFNγ without any effect on the bacterial burden at 24 h post-infection, demonstrating that these factors also regulate IFNγ production during Lm infection. In their study, the authors assumed that NK cells were the main source of IL-10, based on their previous published work [5].

In that study, the levels of serum IL-18 were also found to be increased in parallel with those of serum IL-10 in Lm-infected B6 mice at 72 h post-infection, whereas B6.*Nlrp3^−/−^* mice had significantly reduced serum IL-18, which indicated that NLRP3 expression is relevant for IL-18 production. The source of IL-18 was then determined to be DCs through in vitro experiments, where Lm-infected bone-marrow-derived DCs (BMDCs) produced IL-18, whereas B6.*Nlrp3^−/−^* BMDCs failed to release IL-18. The authors acknowledged that there are diverse cell types that may produce IL-18 that were not additionally tested. Evidence showed that supernatants from Lm-infected B6 BMDCs promoted NK cell IL-10 production, whereas supernatants from B6.*Il18^−/−^*, B6.*Nlrp3^−/−^*, and B6.*Batf3^−/−^* BMDCs did not. Recombinant IL-18 restored the ability of supernatants from B6.*Il18^−/−^* BMDCs to promote NK cell IL-10 production. However, the addition of recombinant IL-18 alone did not induce NK cell IL-10 production in the absence of conditioned BMDC supernatants. These data demonstrate that BMDC release of IL-18 together with an additional factor (or factors) elicit IL-10 production by NK cells. Any additional factors are not dependent on NLRP3 because the B6.*Nlrp3^−/−^* BMDC supernatants were able to promote IL-10 production by NK cells when recombinant IL-18 was added to the culture.

These data also verify that IL-18R is required by NK cells to produce IL-10 through in vitro experiments with B6.*Il10^−/−^* BMDCs infected with Lm and cultured with purified splenic B6 or B6.*Il18r1^−/−^* NK cells. In contrast to co-cultures with B6 NK cells, no IL-10 was produced in cultures containing only B6.*Il18r1^−/−^* NK cells. In summary, IL-18 produced by *Nlrp3*^+^Batf3^+^ DCs, together with another DC factor(s), promotes IL-10 production by NK cells in Lm systemic infection (Figure 2b).

In 2019, Clark et al. [21] investigated which other factor(s) may be required for IL-10 production by NK cells during Lm systemic infection. Their new approach consisted of a model of conditionally mutant mice lacking Stat3, IL-15Rα, or IL-10Rα selectively in NK cells (NKSTAT3^−^, NKIl10R^−^, and NK15Rα^−^ mice, respectively) to investigate the requirements for NK cell IL-10 production during both Lm infection and IL-15 and IL-15Rα complex (IL-15C) treatment.

First, they showed that STAT3 activation was associated with IL-10 production by NK cells during Lm infection by detecting phosphorylated STAT3 (p-STAT3) in the lysates of NK cells purified from Lm-infected B6 mice at both 24 h and 72 h post-infection. However, the amount of p-STAT3 at 24 h was similar in NK cells from B6 and B6.*Il10^−/−^* mice, indicating that STAT3 activation occurs in NK cells at 24 h and 72 h post-infection, but that early activation is independent of IL-10 production. Using Lm-infected B6, NKSTAT3^−^, and B6.*Il10^−/−^* mice, they then showed that intrinsic STAT3 is required for the IL-10 NK cell response during systemic Lm infection. They found that serum IL-10 levels were increased at 72 h post-infection in infected B6 mice but not in infected NKSTAT3^−^ and B6.*Il10^−/−^* mice, whereas the Lm burdens in the liver and spleen of NKSTAT3^−^ and B6.*Il10^−/−^* mice were significantly reduced. The recruitment of inflammatory myeloid cells to the spleens of Lm-infected mice was increased in infected NKSTAT3^−^ and B6.*Il10^−/−^* mice at 72 h post-infection, which correlates with improved resistance to Lm systemic infection.

Because IL-15C treatment induces IL-10 production by NK cells with a similar kinetics to the one observed during Lm infection, the authors used this model for tests. They found that the expression of IL-10 transcripts was significantly reduced in NK cells from NKSTAT3^−^ compared with B6 mice treated with IL-15C, suggesting that intrinsic STAT3 is also required for the NK cell IL-10 response induced by IL-15C treatment. The authors then tested whether IL-15 directly affects the IL-10 production by NK cells using NK15Rα^−^ mice. They found that levels of serum IL-10 and Lm burdens in the liver and spleen were reduced in Lm-infected NK15Rα^−^ compared with B6 mice, indicating the relevance of the IL-15 receptor to IL-10 production by NK cells. Moreover, NK cells from NK15Rα^−^ and B6 mice had similar amounts of total STAT3, but the amount of p-STAT3 was significantly reduced in the IL-15Rα-deficient NK cells at 72 h post-infection, which indicated that this receptor contributes to the STAT3 activation. Together, the data indicate that IL-15Rα contributes to STAT3 activation and IL-10 production in NK cells during Lm infection.

Finally, the authors examined STAT3 activation, IL-10 production, Lm burden, and inflammatory myeloid cell accumulation in the organs of IL-10Rα deficient-NK cells (NKIl10R^−^) mice at 72 h post-infection with Lm. They found an expected high increase in p-STAT3 in the infected B6 mice, but the p-STAT3 response was weak in NK cells from NKIl10R^−^ mice at 72 h post-infection. Serum IL-10 and Lm burdens in the spleen and liver were significantly reduced, whereas more inflammatory myeloid cells accumulated in the spleens of NKIl10R^−^ mice compared with B6 mice at 72 h post-infection. These data indicated that expression of IL-10Rα by NK cells is critical for boost STAT3 activation up to 72 h post-infection and to stimulate IL-10 production in NK cells during Lm infection. In summary, evidence indicates that IL-15 signaling induces early STAT3 activation in NK cells, which is required for the IL-10 production response to Lm infection, which in turn feeds back through IL-10R on NK cells to promote sustained STAT3 activation and IL-10 production by NK cells observed at 72 h post-infection (Figure 2c). This sustained IL-10 production by NK cells is associated with increased susceptibility to Lm systemic infection. 

### 2.2. IL-10-Producing NK Cells during Experimental Visceral Leishmaniasis

Leishmaniasis comprises a group of diseases caused by an intracellular protozoan of the genus *Leishmania* that are transmitted by the bite of phlebotomine sandflies. Leishmaniasis is a major public health issue, with 350 million people living at risk of developing the disease in 98 countries and with an estimated 20,000–50,000 deaths annually worldwide [22,23]. There are three major forms of the disease, namely, cutaneous, mucocutaneous, and visceral, all of which depend on the infective *Leishmania* species and the host immune response. Visceral leishmaniasis is the more severe form of the disease. This is characterized by parasite dissemination to internal organs, such as the liver, spleen, and bone marrow, with a frequently fatal prognosis without adequate treatment. The causal species for visceral leishmaniasis are *L. donovani* and *L. infantum/chagasi* [22,23]. It is known that protective immune response against *L. donovani* infection consists of a granulomatous inflammatory response that restrains the parasite and is positively regulated by cytokines produced by both Th1 and Th2 and negatively regulated by IL-10 produced by Treg cells [22,23,24,25,26]. 

In 2008, Maroof et al. [6] investigated the immunoregulatory role of NK cells in the murine model of experimental visceral leishmaniasis. They reported that IL-10-producing NK cells migrate into the spleen and hepatic granulomas of *L. donovani*-infected mice and suppress host resistance to disseminated parasitosis, which is dependent on IL-10 production. Similar to what was found in the Lm systemic infection model, NK cells are able to produce IL-10 in the early infection phase, but sustained IL-10 production at the late phase of the infection is associated with the increased susceptibility of the host to the systemic infection. In the BALB/c mouse model of visceral leishmaniasis, the presence of liver granulomas is observed at 14–28 days post-infection. At 28 days post-infection, hepatic resistance becomes detectable (reduction of parasite burden in liver), whereas parasite numbers in the spleen are increasing. The authors first found that NK cells were the main source of IL-10 (mRNA) at 14 days post-infection with *L. donovani* by analyzing the purified leucocyte population from the spleen of infected mice, whereas T CD4^+^ and NK cells were the main sources of IL-10 production at 28 days post-infection. Infected mice were also found to have an accumulation of NK cells expressing IL-10 mRNA within immature and mature granulomas and within liver parenchyma. NK cells with high levels of IL-10 mRNA were also detected in the spleen and liver of infected mice by day 7 post-infection, indicating an early IL-10 production during *L. donovani* infection.

**Figure 3:**
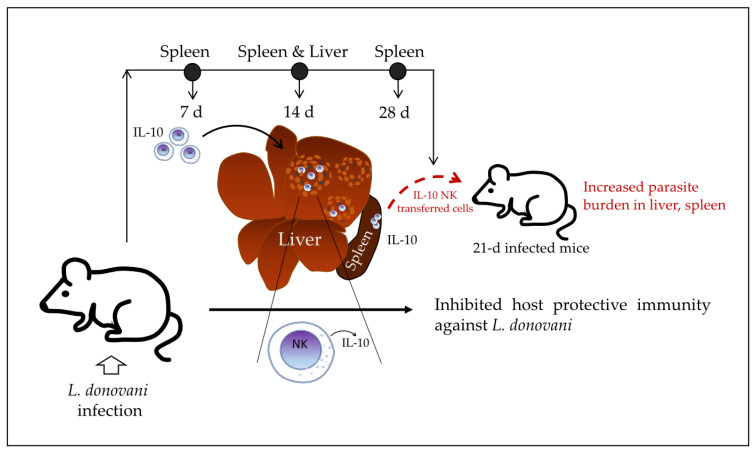
Proposed model of the role of IL-10-producing NK cells in visceral leishmaniasis. Splenic NK cells produce IL-10 from an early stage of infection (7 days post-infection) through late stages (14–28 days post-infection) with *L. donovani*. IL-10-producing NK cells migrate into liver granulomas and spleen (14 days post-infection) and become the main source of IL-10 (14 days and 28 days post-infection) in both organs. IL-10-producing NK cells at late stages (28 days post-infection) induce an increased parasite burden in the liver and spleen of 21-day infected recipient mice when they are transferred, suggesting that they inhibit host protective immunity against *L. donovani*. *L. donovani—Leishmania donovani*; d—days.

To elucidate whether IL-10 produced by NK cells may affect the outcome of the infection, the authors generated CD45.1 B6.IL-10^+/+^ and CD45.2 B6.IL-10^−^^/−^ mice, infected them with *L. donovani*, and then purified splenic NK cells by sorting at 28 days post-infection. The purified NK cells were then transferred into 21-day-infected CD45.1 B6.IL-10^+/+^ recipient mice. They found that the transferred IL-10^+/+^ NK cells increased the parasite burden in the spleen and liver of recipient mice, whereas the transferred IL-10^−/−^ NK cells did not have an effect on the parasite burden of recipient mice compared with control mice without transferred cells. The authors suggested that transferred NK cells with the ability to produce IL-10 may suppress hepatic resistance to *L. donovani* in the infected recipient mice; however, the impact of the IL-10-producing NK cells from the recipient mice themselves cannot be tested in this model. Perhaps a model of IL-10^−/−^ or NK-depleted NK cell recipient mice should be used to test the specific impact of the transferred IL-10^+/+^ NK cells on parasite burden. Together, the data indicate that NK cells produce IL-10 at early stages of visceral leishmaniasis in mice and continue producing IL-10 at a higher concentration over the course of infection while migrating into granulomas in the liver of infected mice. At late stages of infection, these IL-10-producing NK cells may induce an increased parasite burden in 21-day-infected recipient mice, which suggests that they inhibit host protective immunity against *L. donovani* (Figure 3).

### 2.3. IL-10-Producing NK Cells in Experimental Cerebral Malaria (ECM)

In humans, cerebral malaria is a severe complication of *Plasmodium falciparum* infection, leading to a high mortality rate in patients with malaria, mainly young children [27]. The pathogenesis of cerebral malaria is not completely understood, but a consistent characteristic is the sequestration of infected erythrocytes within cerebral blood vessels, which may lead to endothelial damage, disruption of the vessel wall, myelin and axonal damage, and breakdown of the blood–brain barrier [28]. There have been several strategies to elucidate the mechanisms underlying the pathogenesis of cerebral malaria, such as the analysis of clinical case series and case–control studies, post-mortem analysis, and experimental murine models [29]. ECM is an accepted murine model, where several events similar to those occurring in human cerebral malaria are replicated when susceptible mouse strains, such as C57BL/6, are infected with *Plasmodium berghei* ANKA (PbA) [30].

**Figure 4:**
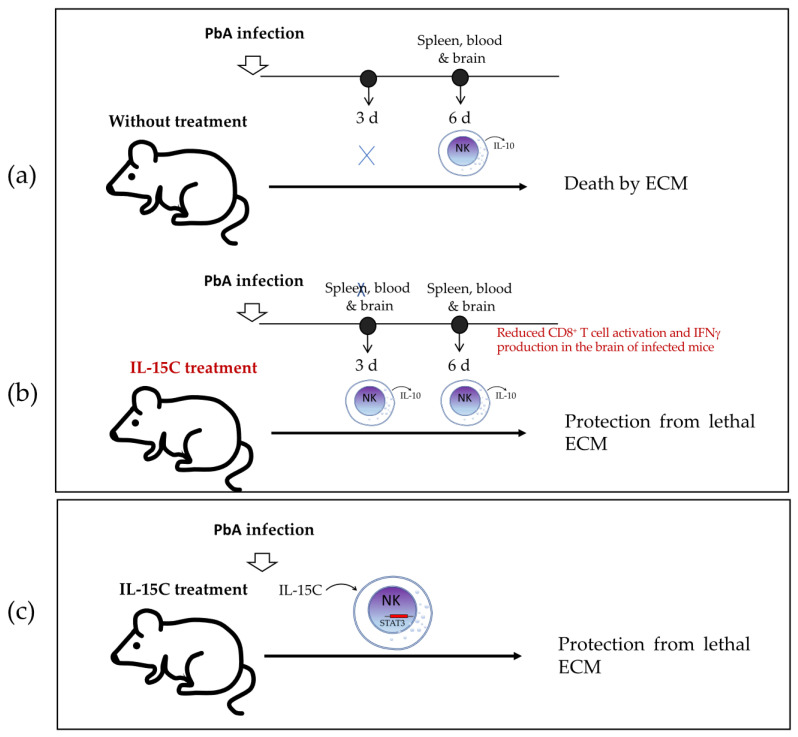
Proposed models of the role of IL-10-producing NK cells in experimental cerebral malaria (ECM). (**a**) NK cells produce IL-10 during ECM in mice infected with PbA at day 6 post-infection, which results in a fatal outcome, whereas (**b**) the treatment with IL-15C promotes an early IL-10 production by NK cells (3 days post-infection), which is sufficient and necessary for the protective effect of IL-15C treatment in preventing the development of ECM and the fatal outcome. In addition, IL-15C treatment decreases CD8^+^ T-cell activation and IFNγ production in the brain of infected mice at 6 days post-infection. (**c**) In this model, NK cell intrinsic STAT3 is required for the survival-promoting effects of IL-15C treatment of ECM. ECM—experimental cerebral malaria; PbA—*Plasmodium berghei* ANKA; d—days; IL15C—IL15 and IL15R complex treatment.

Burrack et al. [8] investigated in 2018 whether cytokine/cytokine receptor complex therapy (with IL-15/IL-15R or IL-2/IL-2R) modulates the immune cell activation in ECM. They reported that only IL-15 complex therapy (IL-15C) prevents mice from death by ECM in PbA-infected C57BL/6 mice, which occurs through stimulation of NK cells to produce IL-10. They first treated C57BL/6 mice with IL-15C or IL-2C prior to PbA infection and found that prophylactic treatment with IL-15C, but not IL-2C, prevented lethal ECM in most of the infected mice. They then used *Il10*-eGFP-reporter mice (VertX, with genetic background C57BL/6J) to determine that on day 3 in the spleen, blood, and brain of infected mice following IL-15C treatment, the cell population within IL-10^GFP+^ cells consisted of a majority of NK cells. IL-10^GFP+^ NK cells were also the main population in the spleen, blood, and brain of untreated mice but only until day 6 post-infection, when there was death from ECM, suggesting that IL-15C treatment induces early IL-10 production that may be related to the protective effect. Furthermore, they demonstrated that IL-10 was necessary for IL-15C-mediated protection from ECM by comparing *Il10*^−/−^ mice with wild-type (wt) mice, the latter of which were protected from PbA infection-induced ECM after IL-15C treatment, whereas *Il10*^−/−^ mice were not. Finally, the authors investigated whether IL-10 production by NK cells is essential for the IL-15C-mediated protection by transferring NK cells from IL-15C-treated wt or *Il10*^−/−^ mice into wt recipients on day 2 post-PbA infection; they found that the transfer of wt NK cells protected against ECM, while most PbA-infected mice receiving *Il10*^−/−^ NK cells died, similar to untreated controls. Because NK cell adoptive transfer had no effect on parasitemia levels, they suggested that the protective role of IL-10 produced by NK cells was related to the development of cerebral malaria but not to the control of the infection itself. In summary, the findings of the study indicated that NK cells produce IL-10 during ECM in mice infected with PbA, but that this induction occurs too late to prevent the fatal outcome (Figure 4a), whereas the treatment with IL-15C promotes early IL-10 production by NK cells that is sufficient to protect mice from the fatal outcome (Figure 4b).

In addition, the authors investigated the effect on CD8^+^ T-cell activation of treatment with IL-15C, which migrates into the brain during the development of ECM. They used flow cytometry, *Nr4a1*^GFP^ reporter mice, GAP50_41-48_/D^b^ tetramers, and *Ifng*^YFP^ reporter mice to evaluate activation of antigen-specific CD8^+^ T cells. *Nr4a1* encodes Nur77, which is expressed in direct proportion to the strength of TCR activation; GAP50 is the glideosome-associated protein 50 from *Plasmodium* that functions as a highly immunogenic CD8^+^ TCR epitope in C57BL/6J mice; and IFNγ is associated with ECM immunopathology. They found that the number of Nr4a1^GFP+^ GAP50-specific CD8^+^ T cells was significantly reduced in the brains of IL-15C-treated mice (6 days post-infection) compared with untreated mice. Using the *Ifng*^YFP^ reporter mice, they found that IL-15C-treated mice had significantly reduced frequencies of *Ifng*^YFP+^ total and GAP50-specific CD8^+^ T cells and reduced *Ifng*^YFP^ mean fluorescence intensity (MFI) compared with untreated mice, although the total number of *Ifng*^YFP+^ cells was not different between the groups. These data indicate that IL-15C treatment reduces CD8^+^ T-cell activation and IFNγ production in the brain of infected mice, although it is not known whether the NK cells that produce IL-10 have any direct role in this reduced T-cell activation (Figure 4b).

Clark et al. [21], in addition to investigating the factors required for NK cell IL-10 production in Lm infection as reviewed in Section 2.1, also evaluated the impact of NK cell intrinsic STAT3 on the protective effect of IL-15C treatment in ECM. They confirmed that IL-15C treatment protects mice against lethal ECM and found that STAT3-deficient NK cells (NKSTAT3^−^) mice succumbed to ECM regardless of receiving IL-15C treatment. These findings indicate that NK cell STAT3 is required for the survival-promoting effects of IL-15C treatment in ECM (Figure 4c).

### 2.4. IL-10-Producing NK Cells during Murine Cytomegalovirus (MCMV) Infection

The human cytomegalovirus (HCMV) is a double-stranded DNA beta-herpesvirus with a high prevalence in the general population. After the establishment of infection, HCMV becomes latent and persists throughout the lifetimes of healthy asymptomatic persons. The reactivation of latent HCMV is usually asymptomatic but results in the production of infectious virions that are transmitted via the saliva, urine, and other body fluids. During active infection of immunocompetent individuals, HCMV is found in the salivary glands, blood, kidneys, and liver. In normal individuals, subclinical hepatitis is frequently associated with HCMV infection, which may be caused by the host inflammatory response or by direct virus cytopathogenicity [31]. Although HCMV infection is usually controlled by the immune system in healthy individuals, it can be life-threatening for immunocompromised patients, such as transplant recipients, AIDS individuals, and neonates [31,32,33]. 

**Figure 5:**
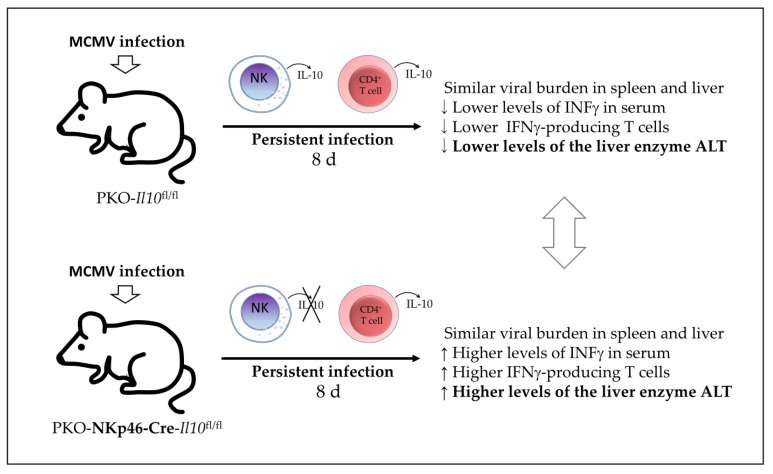
Proposed model of the role of IL-10-producing NK cells in murine cytomegalovirus (MCMV) infection. IL-10-producing NK cells prevent liver damage and potentially regulate production of INFγ in a model of perforin-deficient mice (PKO). PKO mice suffer from persistent MCMV infection, with NK cells and CD4^+^ T cells as the main source of IL-10 at 8 days post-infection. PKO-IL-10-deficient NK cell mice (PKO-NKp46-Cre-Il10^fl/fl^) show increased liver damage (higher levels of the liver enzyme ALT), increased serum levels of INFγ, and INFγ^+^ T cells, but a viral burden similar to the infected PKO control mice (PKO-Il10^fl/fl^). MCMV—murine cytomegalovirus; PKO mice—perforin-deficient mice; ALT—alanine aminotransferase.

Mouse animal models of MCMV infection have been used to investigate the pathogenesis of HCMV and the role of the immune response components because they share several common features. During MCMV infection, the virus replicates in organs such as the spleen and liver, whereas NK cells and macrophages are recruited in the early phases of infection, and pro-inflammatory INFγ production by NK cells is known to function in containing MCMV infection spread [34,35,36]. CD4^+^ T cells and CD8^+^ T cells are subsequently recruited into the infected organs to participate in the pathogen clearance [37,38]. This protective immune response must be regulated to avoid damage to organs, such as the liver [36,39]. Ali et al. [7] investigated in 2019 whether IL-10-producing NK cell have a dual role in the regulation of inflammatory responses during MCMV infection. Using a model of IL-10 GFP-reporter mice, they first found that NK cells in the peripheral blood are the major producers of IL-10 GFP during the early stages of MCMV infection, with a peak observed on day 4 post-infection. Using an NK cell-specific IL-10-deficient mouse (NKp46-Cre-Il10^fl/fl^) and their control Il10^fl/fl^ mice, they found that the presence of NK cell-derived IL-10 did not affect the viral clearance in the spleen, liver damage, and the production of IFNγ by T cells in the spleen of infected mice. Although the authors were able to eliminate IL-10-producing NK cells in the NKp46-Cre-Il10^fl/fl^ mice, the levels of IL-10 were not altered in the serum at day 4 post-infection, indicating another source of IL-10 in this model during acute MCMV infection. Therefore, during acute MCMV infection in these mice, IL-10 production by NK cells is not required for pathogen clearance nor control of the IFNγ-mediated T-cell response. However, the authors tested another model by producing PKO-IL-10 GFP mice and PKO-NKp46-Cre-Il10^fl/fl^ mice and found that perforin-deficient (PKO) mice could not clear the MCMV-infected cells efficiently and subsequently suffered from persistent infection. In this PKO murine model of persistent infection, they found that NK cells and CD4^+^ T cells produced high levels of IL-10 GFP for a longer time (until day 8 post-infection). Meanwhile, infected PKO-NKp46-Cre-Il10^fl/fl^ showed an overall reduction in the serum IL-10 levels and increased serum INFγ levels and liver damage (as determined by increased levels of the enzyme alanine aminotransferase or ALT) compared with their infected control littermates PKOIl10^fl/fl^, although they had similar virus titers in their spleens and livers. Therefore, the authors reported that NK cell-derived IL-10 prevents liver damage and potentially regulates INFγ production in a model of perforin-deficient mice suffering from persistent MCMV infection (Figure 5). However, the potential participation of CD4^+^ T-cell-derived IL-10 in this MCMV infection model was not discussed in this study.

### 2.5. IL-10-Producing NK Cells during Sepsis

The Third International Consensus Definition for Sepsis defines sepsis as a life-threatening organ dysfunction caused by dysregulated host response to infection [40]. Sepsis is a relevant public health problem with a high incidence and is one of the leading causes of death worldwide [41,42]. Sepsis comprises complex immune and physiological responses to systemic infection that are still poorly understood and can lead to organ dysfunction and a high risk of death [43]. It is known that dysfunctional innate and adaptive immunity simultaneously elicit inflammatory and anti-inflammatory responses that can exist synchronously, and the sustained effect leads to organ injury [44]. Over 100 clinical trials targeting the pro-inflammatory component of the cytokine storm in sepsis have failed, indicating that targeting only the pro-inflammatory components is not the right approach for addressing the dualistic/synchronous pro- and anti-inflammatory response in sepsis [45]. NK cells are known for their classic pro-inflammatory function in sepsis, but their dual role as immunoregulatory factors in sepsis has been poorly explored.

Jensen et al. [9] in 2021 investigated the role of IL-10-producing NK cells using a cecal ligation and puncture (CLP) model of sepsis induction. They hypothesized that NK cells may have a dual role as a classic pro-inflammatory and as a novel anti-inflammatory effector during the biphasic inflammatory states of sepsis. The authors used a CLP model where the severity of the septic events was modulated by varying the number of cecal punctures: CLP_50_ (two punctures) and CLP_20_ (one puncture) were performed to evaluate mortality and morbidity in high and low disease severity, respectively. They first depleted the total NK cells from mice using anti-NK1.1-depleting antibodies (depletion that lasted up to 10 days) and found that NK-depleted animals who underwent CLP_50_ had increased mortality and an increased scope of 28 plasma cytokines, compared with NK-replete mice. In addition, the concentration of the three cytokines associated with sepsis severity (IL-6, IL-1β, and IFNγ) was maintained in NK-depleted mice over an extended duration without any increases in magnitude, suggesting a NK cell-limiting effect on the duration and scope of the cytokine storm. Because the authors found an elevated IL-15 concentration in the plasma of NK-depleted mice, they tested the effect of IL-15 on splenocyte cultures, which showed increased IFNγ production during the first 24 h and a subsequent increased IL-10 production by NK cells after 48 h of culture, indicating a time-regulated dual response to IL-15. They then showed that the NK cells of mice that underwent CL_50_ and CLP_20_ had expressed IL-10 mRNA.

**Figure 6:**
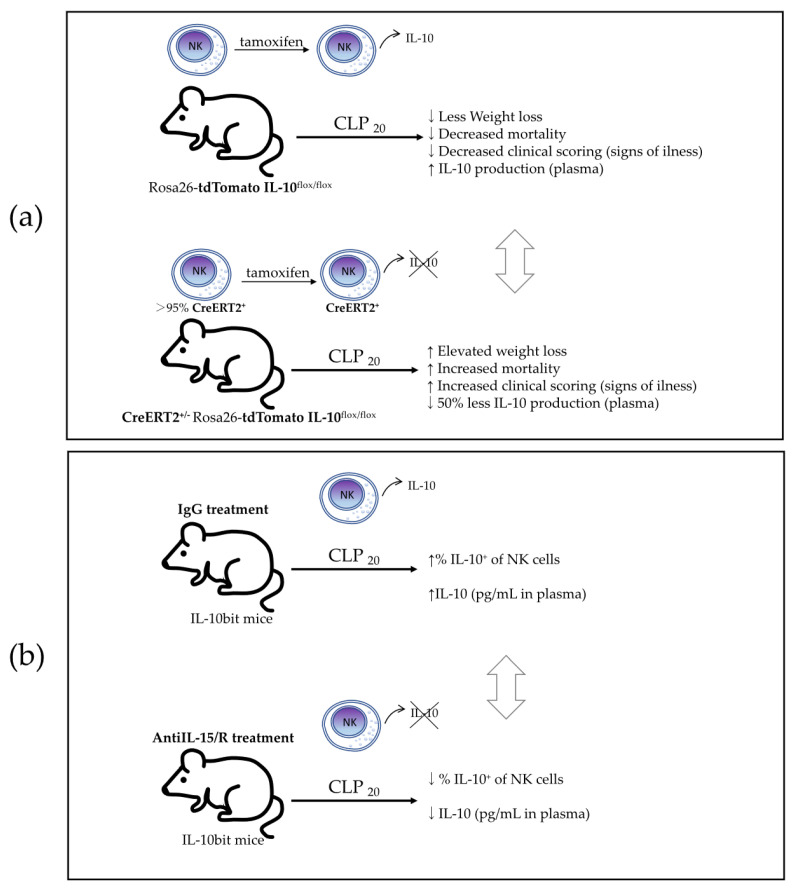
NK cell-derived IL-10 supports host survival during sepsis, which is produced in an IL-15-dependent manner. The role of NK cell-derived IL-10 was investigated by using a cecal ligation and puncture (CLP) model of sepsis induction in mice. (**a**) IL-10-deficient NK cell (NCR1-CreERT2^+/2^ Rosa26-tdTomatoIL-10^flox/flox^) mice and their control (Rosa26-tdTomato IL-10^flox/flox^) littermates were treated with tamoxifen to knock out IL-10 expression in NK cells expressing the CreERT2, and mortality and morbidity were evaluated after CLP_20_. (**b**) IL-10bit mice (which express Thy1.1 during active IL-10 transcription) were treated with either anti-IL-15/R-blocking antibodies or control IgG, and the frequency and number of IL-10-producing NK cells along with plasma IL-10 concentration were measured. CLP_20_—CLP with 1 puncture (as a model of low severity sepsis).

The authors finally proved that NK cell-derived IL-10 is critical for host survival during sepsis using a model of NCR1-CreERT2^+/2^ Rosa26-tdTomato IL-10^flox/flox^ mice along with Rosa26-tdTomato IL-10^flox/flox^ littermates that were treated with tamoxifen to knock out IL-10 expression in NK cells expressing the CreERT2 (Figure 6a). Mice whose NK cells lacked IL-10 (NCR1-CreERT2^+/2^ Rosa26-tdTomato IL-10^flox/flox^) had elevated weight loss, increased mortality, and prolonged signs of illness after undergoing CLP_20_-induced sepsis. Surprisingly, they did not test any cytokines in this model to verify that NK cell-derived IL-10 limits the scope and duration of the cytokine storm, as suggested in their previous study using a model of total NK-depleted mice. Additionally, the authors showed that NK cells produce IL-10 in an IL-15-dependent manner by using IL-10bit mice (which express Thy1.1 during active IL-10 transcription) treated with either anti-IL-15/R-blocking antibodies or control IgG. In the mice treated with anti-IL-15/R-blocking antibodies after they underwent CLP_20_, they found a reduced frequency of IL-10-producing NK cells, but not a reduced frequency or number of total NK cells, and a reduced concentration of plasma IL-10 (Figure 6b), indicating the necessity of IL-15 for IL-10 production by NK cells. To translate these findings to human disease, the authors showed an increased production of IL-10 by NK cells in septic patients, together with an elevated concentration of IL-10, IFNγ, and IL-6 in plasma, compared to healthy controls. They also found that the IL-10 concentration was positively correlated with the IL-6 concentration and negatively correlated with the concentration of IFNγ, suggesting a potential regulation role of the pro-inflammatory signals. This work reported the production of the immunosuppressive cytokine IL-10 produced by NK cells in a murine model of sepsis and in human sepsis disease, revealing a potential immunoregulatory mechanism of NK cells during sepsis that may have a crucial role in the survival of the host.

### 2.6. IL-10-Producing NK Cells during Lung Infections

The airways are one of the primary routes for pathogens to enter the lungs and body and therefore require an effective immune response to prevent pathogens from spreading. However, since the lungs are sensitive organs in which the essential exchange of breathing gases takes place, any inflammatory response must be tightly regulated in order to eliminate pathogens but prevent immune diseases and chronic inflammation [46]. The lungs contain the second highest percentage of NK cells in the body, which play an important role in the inflammatory response to lung infections. NK cells are known to migrate from the blood into the lungs within hours of inflammatory signals and secrete pro-inflammatory cytokines, such as IFNγ, but this inflammatory response is rapidly controlled by IL-10 and transforming growth factor (TGFβ) produced by alveolar macrophages [47]. However, it was unclear whether the NK cells resident in the lungs could play an immunoregulatory role through the production of IL-10.

Clark et al. [48] showed in 2020 that IL-10 produced by NK cells in the lung has a negative immunoregulatory role during sublethal *Streptococcus pneumoniae* (*S. pneumoniae*) infection, promoting bacterial burden in the lung. First, they used tiger IL-10 GFP-reporter mice to determine that NK cells from the blood and lung, but not other lung cells, are the main source of IL-10 after 72 h of intranasal infection with sublethal doses of *S. pneumoniae*. They then performed adoptive transfer experiments with CD45.2^+^ or CD45.1^+^ B6.*il10*^−/−^ recipients that were infected with *S. pneumoniae* and then received NK cells from wild-type (wt) CD45.1 (B6) or from IL-10-deficient CD45.2 (B6.*il10*^−/−^) mice. Donor NK cells were injected directly into the lungs of IL-10-deficient recipient mice infected with *S. pneumoniae* at 24 h post-infection, and bacterial burdens, IL-10, and lung myeloid cell populations were analyzed at 96 h post-infection. Results indicated that compared with recipients of IL-10-deficient NK cells, the mice that received wt NK cells had increased bacterial burdens in the lung but not other systemic tissues. In addition, the total number of neutrophils and alveolar macrophages in mice that received IL-10-deficient NK cells was higher compared with wt NK cells, suggesting that IL-10 produced by NK cells in the lung limits the recruitment of myeloid cells to the lung.

On the other hand, Bortell et al. [49] discovered in 2021 that *S. pneumoniae* infection in the lung together with oral coinfection with Lm increase susceptibility to systemic Lm infection in a manner dependent on IL-10 production by NK cells. The IL-10-deficient NK cell Ncr1-iCre-Il10^fl/fl^ mouse strain and wt B6 mice were infected with Lm peroral (PO) or coinfected with *S. pneumoniae* intratracheal (IT) + Lm PO, and the spleens, livers, and lungs were harvested at 3 days post-infection. Lm burdens in the spleens, livers, and lungs of coinfected Ncr1-iCre-Il10^fl/fl^ mice were significantly lower than in coinfected B6 mice, whereas Ncr1-iCre-Il10^fl/fl^ mice infected with only Lm PO did not show any difference compared with Lm PO-infected wt B6, indicating the necessity of coinfections. The author also found that upon co-infection, the Ncr1-iCre-Il10^fl/fl^ mice significantly increased the number of neutrophils in both spleen and lungs when compared to B6-coinfected mice.

Taken together, data from both studies indicated that NK cells are the main source of IL-10 in the lung during *S. pneumoniae* infection, which has a negative immunoregulatory role, promoting susceptibility to *S. pneumoniae* dissemination in the lung and oral Lm coinfections.

## 3. Molecular Mechanisms Involved in Regulation of IL-10 Production by NK Cells during Systemic Infection

### 3.1. Cytokines and Their Transcription Factors Regulating the IL-10 Production by NK Cells

The molecular mechanisms that regulate IL-10 production are relevant to elucidate the pathogenesis of inflammatory diseases and to identify new therapeutic interventions.

**Figure 7:**
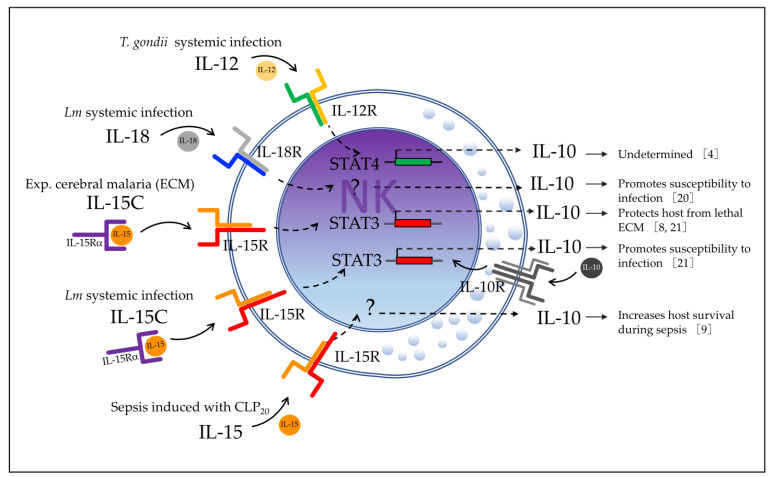
Cytokines and transcription factors that regulate IL-10 production by NK cells in models of systemic infection. Shown are the cytokines and transcription factors associated with induction of IL-10 by NK cells and the effect on the host immunity in the models of *T. gondii* infection, Lm infection, ECM, and sepsis induced with CLP_20_. *T. gondii*—*Toxoplasma gondii*; Lm—Listeria monocytogenes; IL-15C—IL-15 and IL15Ra complex treatment; ECM—experimental cerebral malaria; CLP_20_—cecal ligation and puncture with 1 puncture.

Studies with murine models of systemic infection have shown the association of IL-12, IL-15, and IL-18 cytokines, and some of their transcription factors, with the induction of IL-10 by NK cells (Figure 7). In the model of *Toxoplasma gondii* (*T. gondii*) systemic infection, Perona-Wright et al. [4] showed that production of IL-10 by NK cells was inhibited by anti-IL-12p40 antibody treatment in mice infected with *T. gondii* at 1 week after infection. In addition, they showed that NK cells from IL-12-deficient IL-12Rβ2^−/−^ mice failed to produce IL-10 in *T. gondii*-infected mice compared with control mice. Analysis of ex vivo isolated NK1.1^+^ cells from *T. gondii*-infected mice at 5 days post-infection revealed that signal transducer and activator of transcription 4 (STAT4) was phosphorylated, indicating activation of STAT4 by IL-12 in NK cells. Therefore, IL-12 signal and the activation of STAT4 in NK cells induces IL-10 production in this model.

As described in Section 2.1., Clark et al. [20] reported that systemic Lm infection induces the production of IL-18 by Batf3-dependent DCs that express NLRP3, and that IL-18, in turn, induces NK cells to produce IL-10 via IL-18R. They also found that IL-10 production by NK cells is associated with an immune-suppressive effect on the infected host (Figure 2b and Figure 7). NLRP3 is an intracellular sensor component of a multiprotein complex, called the inflammasome, that recognizes a wide range of microbial motifs and environmental stimuli. The assembly of the NLRP3 inflammasome leads to a caspase-1-dependent release of IL-18. Meanwhile, Batf3 belongs to the AP-1 family transcription factor that controls the differentiation of CD8^+^ thymic conventional DCs. In mice, lymphoid resident Batf3-dependent DCs are characterized by expression of CD8α, whereas Batf3-dependent tissue-resident and migratory lymphoid DCs are instead CD103^+^CD11b^−^. Therefore, the authors revealed an inflammatory-dependent mechanism in which IL-10-producing NK cells play an immunoregulatory role that dampens the host’s resistance to Lm infection. This study also suggested that there is another factor(s) that may regulate the IL-10 production by NK cells. The authors continued this study and published another in 2019 [21] that was described in detail in Section 2.1, in which they reported that IL-15 induces early STAT3 activation in NK cells which is necessary for IL-10 production during systemic Lm infection. This early IL-10 induces sustained STAT3 activation in NK cells via IL-10R with subsequent sustained IL-10 production, which is associated with increased susceptibility to Lm infection (Figure 2c and Figure 7). In this study, the author additionally reported that NK cell STAT3 expression is required for the survival-promoting effects of IL-15C (IL-15 and IL-15Rα complex) treatment of ECM (Figure 4a and Figure 7). 

As described in Section 2.5, Jensen et al. [9] also found that IL-15 and, likely, IL-15R (using anti-IL-15/IL-15R antibody treatment) are required for IL-10 production by NK cells in a CLP_20_-induced sepsis model; however, the role of STAT3 was not investigated in this study (Figure 7).

### 3.2. Surface Receptors Regulating the IL-10 Production by NK Cells

**Figure 8:**
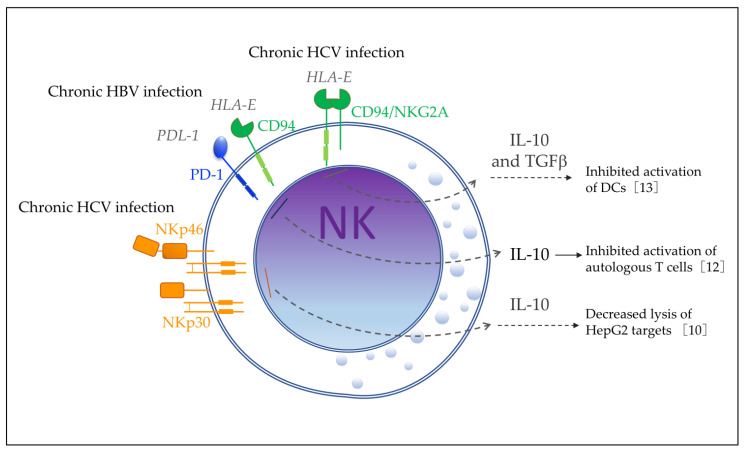
Figure shows the surface receptors associated with regulation of IL-10 production by NK cells and the potential effect on the host immune response in chronic viral diseases. HCV—hepatitis C virus; HBV—hepatitis B virus; DCs—dendritic cells; HepG2—hepatic cell line.

NK cell receptors can be classified according to their structure into the immunoglobulin superfamily (Ig-SF) and the C-type lectin superfamily (CL-SF). They also can be classified according to their function as inhibitory receptors, such as KIR-2DL, KIR-3DL, CD94/NKG2A, PD-1, and TIGIT, and activating receptors, such as KIR-2DS, KIR-3DS, NCR (NKp46, NKp44, and NKp30), NKG2D, 2B4, CD226, and CD94/NKG2C [50]. Some studies have reported that inhibitory receptors are associated with NK cells that produce IL-10 in chronic systemic diseases. Jinushi et al. [13] reported in 2004 that NK cells from HCV patients (HCV-NKs) showed higher expression of CD94/NKG2A inhibitor receptors compared with NK cells from healthy donors (N-NKs). These HCV-NKs were unable to activate DCs when co-cultured with human liver epithelial cells expressing HLA-E, unlike their counterpart N-NKs. The blockade of NKG2A restored the ability of HCV-NKs to activate DCs, suggesting an association with such a function. However, the authors did not provide direct evidence that HCV-NKs produces IL-10; instead, they treated the supernatant of the culture with HCV-NKs/human liver epithelial cells with neutralizing antibodies against IL-10 and TGFβ and found that the NK capacity to activate DCs was restored. Therefore, they indirectly assumed that HCV-NKs produced IL-10 and TGFβ during the co-culture experiments, which may be associated with the inability to activate DCs (Figure 8).

Meanwhile, Li H. et al. [12] found in 2018 that NK cells from chronic hepatitis B virus patients (HBV-NKs) showed higher expression of PD-1 and CD94 inhibitor receptors and higher levels of IL-10 compared with NK cells from healthy donors (N-NKs). They found evidence that such IL-10-producing NK cells inhibited autologous T-cell activation and that IL-10 production was mediated by PD-L1/PD-1 and HLA-E/CD94 interactions with HBV-treated monocytes (Figure 8).

On the other hand, De Maria et al. [10] reported in 2007 that HCV-NKs from chronic HCV-infected patients had selective increased expression of activating receptors of the NCR family NKp30 and NKp46, but not NKG2D, compared with healthy donors (N-NKs). They found that freshly isolated HCV-NKs produced IL-10, whereas N-NK did not. In co-culture experiments, HCV-NKs showed decreased lysis of HepG2 targets, which was associated with NKG2A/CD94 expression in these cells, but evidence of the role of IL-10 produced by NK cells was not provided (Figure 8). The discrepancy between the type of receptors reported in this study and those in the study by Jinushi et al. was due to De Maria et al. only investigating four activating receptors (NKp30, NKp46, NKp44, and NKG2D), whereas Jinushi et al. tested KIRs receptors (KIR2DL1/2DS1, KIR2DL2/2DS2, KIR2DS4, and KIR3DL1) and the activating receptor NKG2D. Neither study reported any difference in the expression of NKG2D in HCV-NKs compared to NKs from healthy donors.

## 4. Role of IL-10-Producing NK Cells in Human Disease

Two studies reported that HCV-NKs from chronically infected viremic patients produce IL-10, which may be associated with a decreased ability to clear the virus. The study by Jinushi et al. [13] investigated the ability of NK cells to modulate DCs activation during chronic HCV infection. As described in Section 3.2, they first found that NK cells from healthy donors (N-NKs) were able to activate DCs when co-cultured with the human liver epithelial cell line Hep3B that expresses HLA-E, but this capacity was not shown by NK cells from HCV-infected patients (HCV-NKs), which expressed higher levels of the inhibitory receptors CD94/NKG2A. Thus, a culture supernatant of HCV-NKs/Hep3B cells was treated with neutralizing antibodies against IL-10 and TGFβ and used for the stimulation of DCs, which enhanced the expression of activation markers CD40, CD86, CD83, and CCR7 on DCs. This result suggested that IL-10/TGFβ production could interfere with DC activation; however, the IL-10 production was not directly tested in HCV-NKs obtained ex vivo or in the culture. Therefore, further studies may be performed to relate the IL-10 produced by NK cells to the diminished capacity to activate DCs in this chronic disease. The second study by De Maria et al. [10], also discussed in Section 3.2, reported that freshly isolated HCV-NKs produce IL-10, whereas N-NKs do not. In this study, HCV-NK cells showed decreased cytotoxic capacity in lysing HepG2, which was associated with the higher expression of NKG2A/CD94 in these cells; however, the role of IL-10 produced by HCV-NKs was not further explored (Figure 8).

In 2009, Brockman et al. [11] analyzed the IL-10 mRNA expression levels in several populations of purified peripheral mononuclear cells from 10 HIV-infected individuals and 9 uninfected controls. They reported that CD14^+^ monocytes were a major source of IL-10 in HIV-infected individuals, but a significant elevation in comparison with controls was also found in CD4^+^ T cells, CD8^+^ T cells, CD19^+^ B cells, and CD56^+^ NK cells. However, the role of IL-10-producing cells or NK cells was not investigated further in this study (Figure 8).

In 2018, Li H. et al. [12] found that monocytes and HBV-NKs from infected patients were the main source of IL-10 and expressed higher levels than those from healthy donors. NK cells and monocytes from healthy donors were co-cultivated in the presence of HBV, and NK cells expressed a higher level of IL-10 than cultures without HBV, which required direct cell-to-cell contact with monocytes. The authors found that monocytes from HBV-infected patients showed increased expression of PD-L1 and HLA-E whereas HBV-NKs from infected patients showed increased expression of PD-1 and CD94 compared with healthy controls. They then performed the blockade of PD-L1/PD-1 and HLA-E/CD94 during the co-culture experiments (NK/monocytes/HBV), which significantly inhibited IL-10 expression in NK cells but increased the IFNγ expression. These results suggested that HBV induces monocytes to promote IL-10 production by NK cells, and this HBV-induced IL-10 production is mediated by the PD-L1/PD-1 and HLA-E/CD94 interaction. They finally investigated whether HBV-induced IL-10-producing NK cells inhibit activation of autologous T cells by culturing autologous T cells with NK cells previously purified from co-cultured monocytes/NKs with or without HBV. T-cell proliferation and IFNγ were measured by flow cytometry and ELISA, respectively. The results showed that HBV/monocyte/NK cell co-cultures suppressed the proliferation of T cells and IFNγ production compared to monocyte/NK cell co-cultures, and when IL-10 was blocked by antibodies, T-cell proliferation and IFNγ production was restored. These findings suggested that HBV induces immunoregulatory monocytes that promote IL-10 production by NK cells, which inhibits T-cell activation (Figure 8).

In 2021, Jensen et al. [9] investigated whether IL-10-producing NK cells were present in septic patients exhibiting severe disease. They examined NK cells from peripheral blood and found an increased production of IL-10 by NK cells in septic patients compared with healthy controls. They also found that IL-10, IFNγ, and IL-6 were all elevated in the plasma of septic patients. In addition, the concentration of plasma IL-10 correlated with the concentration of IL-6, suggesting that IL-10 is produced to regulate the inflammatory response typically mediated by IL-6 in sepsis. No further evidence was provided regarding the role of IL-10-producing NK cells in sepsis.

## 5. Conclusions and Perspective

Publications regarding the function of these regulatory IL-10-producing NK cells are still few, especially in the context of human disorders. Understanding the factors that drive IL-10 production by NK cells and the relevance of their role as immunoregulators may provide leads for therapeutic advancement, particularly in those diseases where infections are not controlled or resolved by the host, or where an uncontrolled systemic inflammatory response may induce immunopathology or death.

Here, we described the available published data regarding the potential immunoregulatory role of IL-10-producing NK cells in systemic infection diseases, mainly in animal models. Altogether, evidence indicates that NK cells indeed act as innate inflammatory effectors during early stages of infections (represented by the IFNγ production), and they acquire the immunoregulatory capacity later during the infection (through IL-10 production), potentially when such infection has been disseminated. Thus, NK function capacity is temporally regulated. Several factors that regulate the IL-10 production by NK cells were revealed, such as cytokines IL-12, IL-15, IL-18, and IL-10 itself, which are produced by DCs, monocytes, NK cells, and other unknown cell sources. Transcriptional factors, such as STAT3 and STAT4, and surface receptors, such as PD-1 and CD94, are also associated with IL-10 regulation. However, the effect of these factors on IL-10 production depends specifically on the type of infection model or disease analyzed. Importantly, the impact of IL-10 production by NK cells on the control and clearance of the pathogen during infection seems to depend on the type of infection model, which was found to be detrimental for the host in murine Lm infection, murine visceral leishmaniasis, and human chronic HBV infection but beneficial for the host in for murine MCV infection, murine ECM, and murine sepsis.

Therefore, the molecular mechanisms that induce the immunoregulatory function of NK cells should be dissected in the context of specific systemic infection models or diseases, the timing of IL-10 release by NK cells (early or later during the infection), and the impact on the host immune response against pathogens.

Additionally, the anatomical site where the NK cells act, such as the lung, should also be included as a determining factor in the impact on the host. As mentioned earlier, the lungs contain the second highest percentage of NK cells in the body, which play an important role in the inflammatory response. However, this is a highly specialized organ that is particularly sensitive to tissue damage if inflammation is not controlled. Therefore, NK cells must balance their functions as an inflammatory effector to control any lung infection and as an immunoregulator to prevent immunopathology.

In conclusion, the dualistic function of NK cells reflects the complexity of the immune response as a whole, and the essential requisite for immune regulation to protect from pathogens without causing substantial damage to the host. Therefore, the classic concept of NK cells as innate inflammatory effectors is no longer accurate. Future research should be directed toward the study of the immune response components as an interactive network, where the balance of inflammatory and anti-inflammatory responses work in a synchronous way. NK cells are a relevant part of the complex regulatory mechanisms involved in homeostasis and immune disease, and their role and specific effect in immune responses should be further investigated.

## Data Availability

No new data were created or analyzed in this study. Data sharing is not applicable to this article.

## Abbreviations

ALT	Alanine aminotransferase
Batf3	Basic leucine zipper transcriptional factor ATF-like 3
BMDCs	Bone-marrow-derived DCs
B6	C57BL/6J mice
B6.*il10*^−/−^	IL-10-deficient CD45.2 mice
CLP	Cecal ligation and puncture
CLP_20_	Cecal ligation and one puncture
CLP_50_	Cecal ligation and two punctures
CL-SF	C-type lectin superfamily
DCs	Dendritic cells
ECM	Experimental cerebral malaria
GAP50	Glideosome-associated protein 50 from *Plasmodium*
HBV	Hepatitis B virus
HBV-NKs	NK cells from chronic HBV patients
HCMV	Human cytomegalovirus
HCV	Hepatitis C virus
HCV-NKs	NK cells from chronic HCV patients
HepG2	Hepatic cell line
Hep3B	Human liver epithelial cell line that expresses HLA-E
HIV	Human immunodeficiency virus
IFNγ	Interferon gamma
Ig-SF	Immunoglobulin superfamily
IL-1β	interleukin-1 beta
IL-6	Interleukin-6
IL-10	Interleukin-10
IL-10R	Interleukin-10 receptor
IL-12	Interleukin-12
IL-12R	Interleukin-12 receptor
IL-15	Interleukin-15
IL-15R	Interleukin-15 receptor
IL-15Rα	Interleukin-15 receptor alpha
IL-15C	IL15 and IL15R complex treatment
IL-18	Interleukin-18
IL-18R	Interleukin-18 receptor
IL-18R1	Interleukin-18 receptor 1
Lm	*Listeria monocytogenes*
*L. donovani*	*Leishmania donovani*
MCMV	Murine cytomegalovirus
MFI	Mean fluorescence intensity
NK	Natural killer
N-NKs	NK cells from healthy donors
NKp46-Cre-Il10^fl/fl^	NK cell-specific IL-10-deficient mouse
NKSTAT3^−^	STAT3-deficient NK cells
NKIl10R^−^	IL-10Rα deficient-NK cells mice
NLRP3	NOD-, LRR-, and pyrin domain-containing protein 3
PbA	*Plasmodium berghei* ANKA
PKO	Perforin-deficient mice
PKO-NKp46-Cre-Il10^fl/fl^	PKO-IL-10-deficient NK cell mice
PKO-Il10^fl/fl^	PKO control mice
PO	Peroral
STAT3	Signal Transducer and Activator of Transcription 3
STAT3-p	Signal Transducer and Activator of Transcription 3 phosphorylated
STAT4	Signal Transducer and Activator of Transcription 4
*S. pneumoniae*	*Streptococcus pneumoniae*
TGFβ	Transforming growth factor beta
*T. gondii*	*Toxoplasma gondii*
*Y. pestis*	*Yersinia pestis*
wt	Wild type
